# Supplementary material for: Autophagy inhibition prevents lymphatic malformation progression to lymphangiosarcoma by decreasing osteopontin and Stat3 signaling
Source: Nat Commun. 2023 Feb 22;14:978. doi: 10.1038/s41467-023-36562-5 (PMC9946935; doi:10.1038/s41467-023-36562-5)
Supplement: Supplementary file 1 — Supplementary Information [file 41467_2023_36562_MOESM1_ESM.pdf]

Supplementary Information

Autophagy inhibition prevents lymphatic malformation progression to lymphangiosarcoma by decreasing osteopontin and Stat3 signaling

Fuchun Yang[1], Shiva Kalantari[1], Banzhan Ruan[1], Shaogang Sun[1], Zhaoqun Bian[2] and Jun-Lin Guan[1*]

[1]Department of Cancer Biology, University of Cincinnati College of Medicine, Cincinnati, OH 45267

[2]Department of Surgery, University of Cincinnati College of Medicine, Cincinnati, OH 45267

*Correspondence: guanjl@uc.edu

Supplementary Figure 1

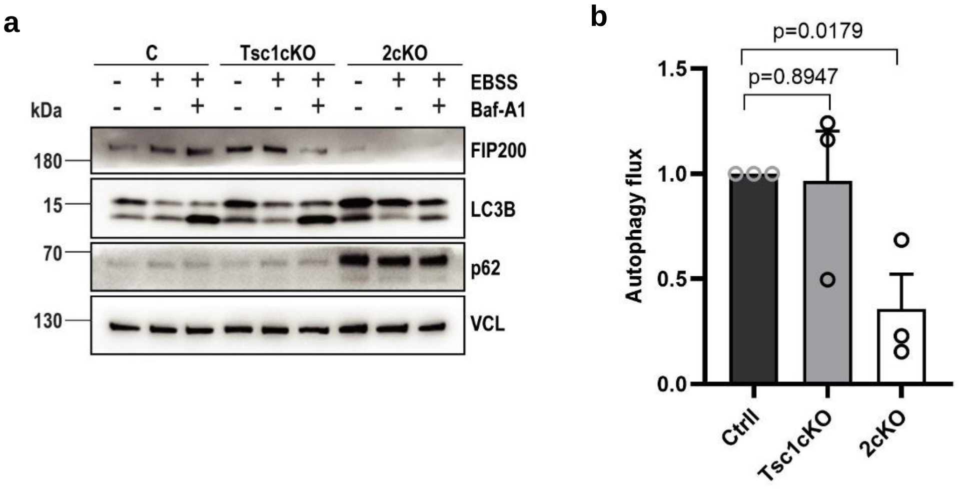

Supplementary Figure 1. Analysis of autophagy flux in lung ECs from various mice. Lung ECs of wild type (C), *Tsc1*$^{i\Delta EC}$ mice and 2cKO mice were incubated in regular EC culture medium without serum (-), EBSS starvation without serum for 4 hours with or without addition of 200 nM Bafilomycin A1 (Bal-A1) for the last 2 hours. Lysates were then prepared and analyzed by western blots for FIP200, LC3B, p62, and vinculin, representative image was shown in **a**. Relative levels of autophagy flux (normalized to Ctrl cells) are shown as mean ± SD in **b** (n = 3 independent experiments). Unpaired two-tailed t test was used in **b**, ns: no significance. Source data are provided as a Source Data file.

Supplementary Figure 2

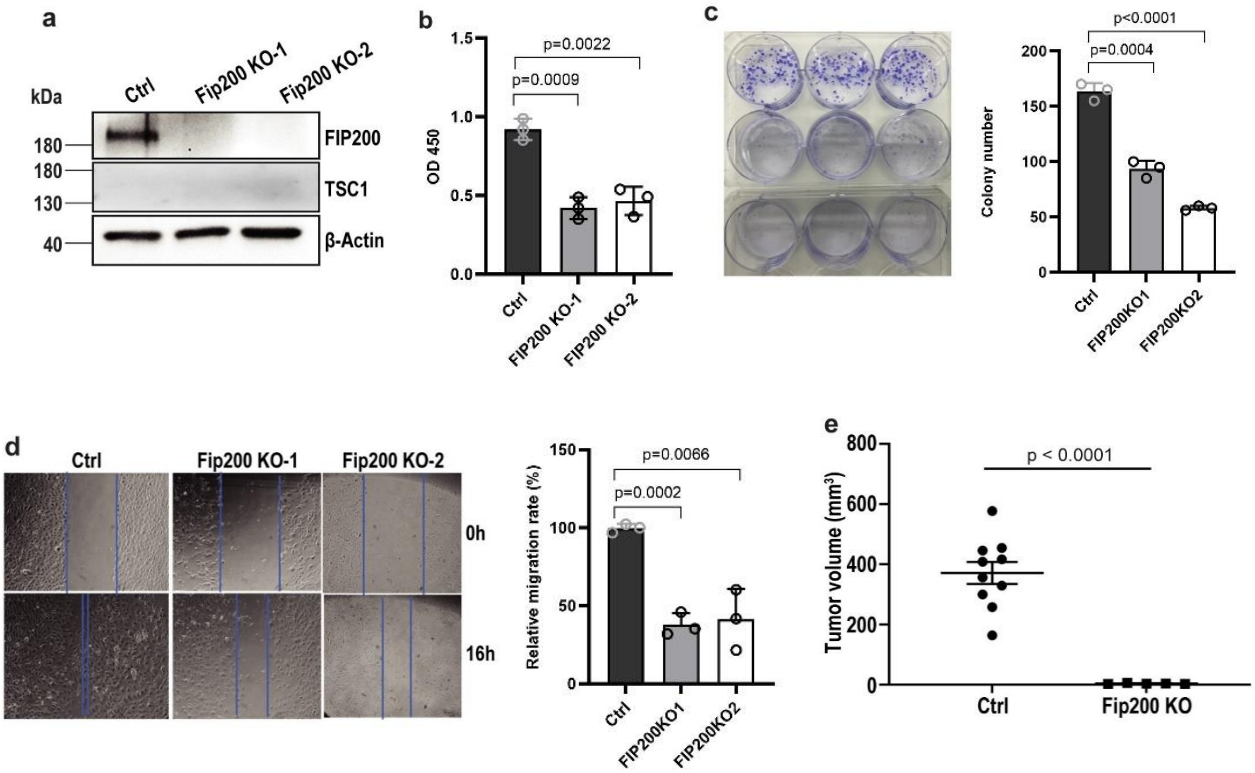

Supplementary Figure 2. Analysis of cellular functions and tumorigenicity of 5864 vascular tumor cells upon FIP200 KO.

a) Lysates from 5864 tumour cells (Ctrl) and two clones of these cells with CRISPR-Cas9 mediated *Fip200* knockout (KO) were examined by western blots for FIP200. TSC1 and b-actin.

b) Ctrl and Fip200 KO tumour cells were measured for cell proliferation by CCK-8 assay shown as mean ± SD. n = 4 biologically independent cells.

c) Representative images of colony formation assay of Ctrl and Fip200 KO tumour cells. Mean ± SD of the colony number is shown on the right. n = 3 biologically independent cells.

d) Representative images of cell migration assay of Ctrl and Fip200 KO tumour cells. Mean ± SD of the relative migration rate is shown on the right. n=3 biologically independent cells.

e) Ctrl and Fip200 KO tumour cells (2x 10^6 cells) were subcutaneously injected in recipient nude mice. Mean ± SEM of tumour volume at 3 months after injection are shown (n = 10 biologically independent samples, 5 mice). Unpaired two-tailed t tests were used in **b, c** (right panel), **d** (right panel), and **e**. For **b-d**, at least two independent experiments were repeated. Source data are provided as a Source Data file.

Supplementary Figure 3

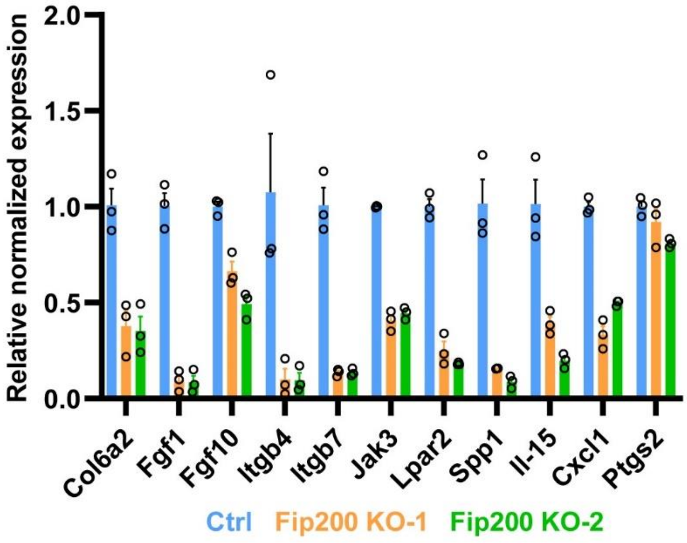

Supplementary Figure 3. Reduced expression of Spp1 and other genes in FIP200 KO tumor cells.

A select group of down-regulated genes including *Spp1* in Fip200 KO cells were validated using RT–qPCR. Mean ± SEM of relative levels (normalized to Ctrl cells) is shown. n = 3 technical replicates. Source data are provided as a Source Data file.

Supplementary Figure 4

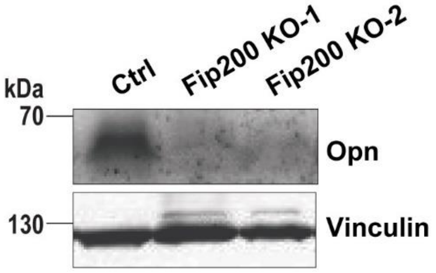

Supplementary Figure 4. Reduced OPN expression in FIP200 KO tumor cells.

Lysates from Ctrl and Fip200 KO tumour cells were examined by western blots for OPN and Vinculin. Source data are provided as a Source Data file.

Supplementary Figure 5

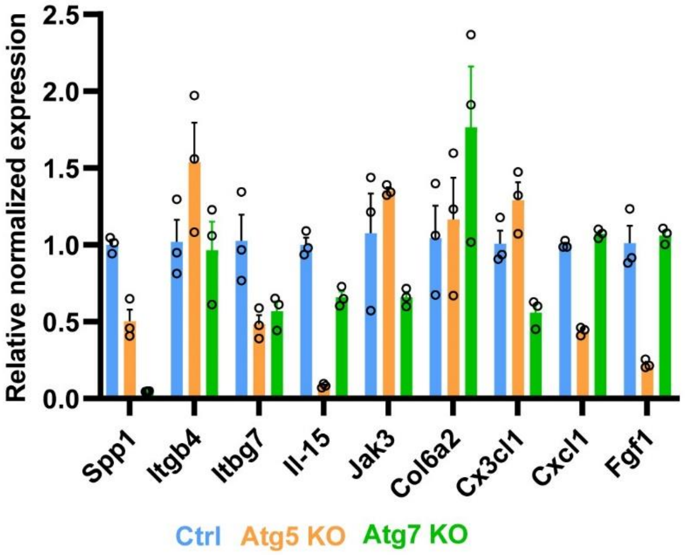

Supplementary Figure 5. Reduced expression of Spp1 and other genes in Atg5 and Atg7 KO tumor cells.

A select group of down-regulated genes including *Spp1* in Atg5 KO and Atg7 KO cells were validated using RT–qPCR. Mean ± SEM of relative levels (normalized to Ctrl cells) is shown. n = 3 technical replicates. Source data are provided as a Source Data file.

Supplementary Figure 6

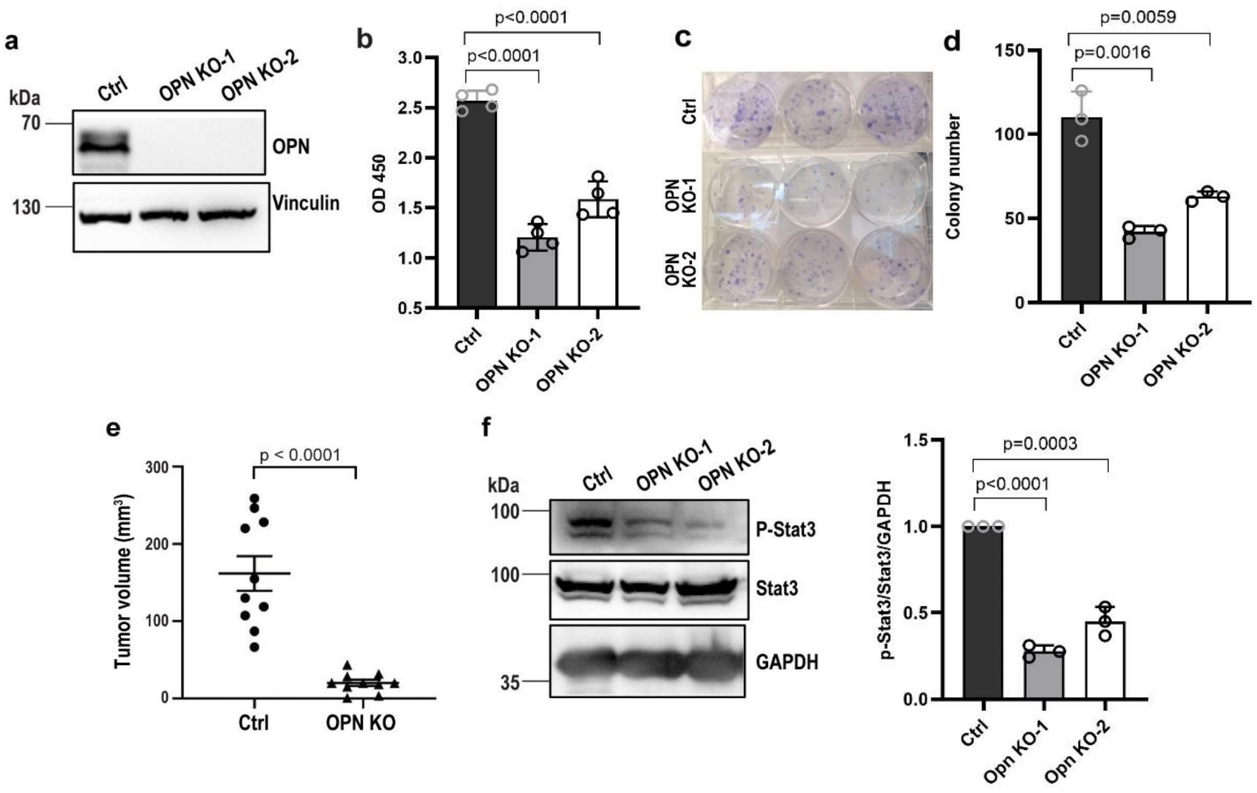

Supplementary Figure 6. Analysis of cellular functions and tumorigenicity of 5864 vascular tumor cells upon OPN KO.

a) Lysates from 5864 tumour cells (Ctrl) and two clones of OPN KO from these cells were examined by western blots for OPN and Vinculin.

b) Ctrl and OPN KO tumour cells were measured for cell proliferation by CCK-8 assay shown as mean ± SD. n = 4 biologically independent cells.

c, d) Representative images of colony formation assay of Ctrl and OPN KO tumour cells are shown in **c**, and mean ± SD of the colony numbers are shown in **d**. n = 3 biologically independent cells.

e) Ctrl and OPN KO tumour cells (2x 10^6 cells) were subcutaneously injected in recipient nude mice. Mean ± SEM of tumour volume at 4 weeks after injection is shown (n = 10 biologically independent samples, 5 mice).

f) Lysates from Ctrl and OPN KO tumour cells were examined by western blots for P-Stat3, Stat3 and GAPDH. Relative levels of pSTAT3/STAT3 (normalized to Ctrl cells) are shown on the right as mean ± SD (n = 3 independent experiments). For **b-d**, at least two independent experiments were repeated. Source data are provided as a Source Data file.

Supplementary Figure 7

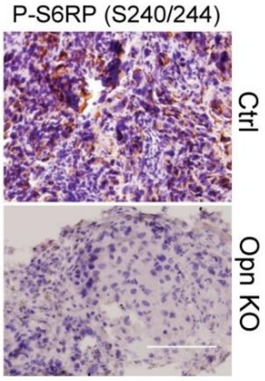

Supplementary Figure 7. Analysis of phospho-S6RP in transplanted tumors from OPN KO cells.

562 tumour cells and OPN KO cells (2X 10^6 cells) were subcutaneously injected in recipient nude mice. Tumors were harvested at 10 weeks after transplantation, and tumor sections were analyzed by IHC for P-S6RP (S240/244). Scale bar, 50 µm.

Supplementary Table 1. Gene list that are common to the top 3 KEGG pathways

	Fip200 KO vs Ctrl		Atg5 KO vs Ctrl		Atg7 KO vs Ctrl	
**Gene**	**log2FC**	**padj**	**log2FC**	**padj**	**log2FC**	**padj**
**Col6a2**	-10.39	2.31E-05	-9.06	1.06E-05	-9.55	4.49E-05
**Cx3cl1**	-2.57	1.08E-04	-2.20	1.12E-04	-1.78	1.91E-02
**Cxcl1**	-2.88	1.24E-02	-3.35	3.63E-04	-3.42	2.26E-04
**Fas**	-2.15	5.58E-02	-2.20	1.03E-03	-2.70	1.40E-03
**Fgf1**	-3.99	6.06E-03	-3.38	1.14E-03	-3.26	6.39E-03
**Fgf10**	-3.34	8.26E-02	-4.30	6.42E-04	-3.41	1.64E-02
**Flt1**	-3.37	4.87E-03	-1.34	3.86E-02	-3.00	1.12E-06
**Gng8**	-5.91	3.33E-02	-5.18	9.33E-03	-5.74	1.26E-02
**Il15**	-3.14	1.09E-07	-3.43	8.37E-21	-1.91	2.79E-03
**Itgb4**	-3.35	1.04E-03	-2.30	2.18E-04	-3.32	1.42E-05
**Itgb5**	-3.42	5.70E-02	-4.37	1.62E-05	-4.66	3.74E-06
**Itgb7**	-4.55	4.35E-04	-5.11	4.24E-07	-4.58	3.36E-04
**Itgb8**	-5.49	4.68E-02	-7.38	1.91E-03	-5.37	1.77E-02
**Jak3**	-1.55	1.53E-02	-2.57	2.17E-13	-1.88	3.83E-04
**Junb**	-1.85	7.58E-03	-1.86	1.03E-04	-1.56	1.30E-02
**Lamb3**	-3.15	6.64E-03	-4.10	1.10E-07	-2.85	1.39E-03
**Lif**	-5.45	8.11E-05	-6.58	2.12E-08	-5.66	6.94E-06
**Lpar2**	-2.04	3.59E-02	-1.57	4.47E-03	-2.62	1.46E-04
**Map3k5**	-3.97	6.24E-03	-3.75	6.08E-04	-4.05	7.07E-04
**Nr4a1**	-2.88	5.20E-05	-2.18	2.08E-05	-2.41	5.31E-04
**Pdgfc**	-3.29	5.92E-02	-3.16	2.84E-04	-3.88	9.05E-05
**Pdgfra**	-3.96	4.90E-03	-4.58	2.50E-04	-4.31	5.21E-04
**Pik3r2**	-1.01	8.81E-02	-1.28	1.38E-05	-1.24	4.06E-03
**Ptgs2**	-1.89	2.41E-02	-3.58	9.75E-14	-1.67	4.26E-03
**Sdc1**	-4.96	3.96E-03	-5.01	2.48E-06	-1.21	8.41E-03
**Sgk1**	-1.56	6.24E-03	-1.37	5.89E-06	-1.07	3.27E-03
**Spp1**	-3.71	1.48E-03	-5.79	3.31E-08	-6.04	3.99E-08
**Thbs2**	-4.84	8.20E-02	-3.28	8.09E-02	-5.69	1.25E-02
**Traf1**	-1.65	1.36E-02	-1.42	5.21E-05	-2.28	5.97E-07
**Vegfd**	-2.80	9.81E-02	-3.39	1.83E-03	-2.59	6.35E-02
