# Supplementary material for: Autophagy inhibition prevents lymphatic malformation progression to lymphangiosarcoma by decreasing osteopontin and Stat3 signaling
Source: Nat Commun. 2023 Feb 22;14:978. doi: 10.1038/s41467-023-36562-5 (PMC9946935; doi:10.1038/s41467-023-36562-5)
Supplement: Supplementary file 3 — Reporting Summary [file 41467_2023_36562_MOESM3_ESM.pdf]

nature portfolio

Corresponding author(s): Jun-Lin Guan
Last updated by author(s): Dec 19, 2022

Reporting Summary

Nature Portfolio wishes to improve the reproducibility of the work that we publish. This form provides structure for consistency and transparency in reporting. For further information on Nature Portfolio policies, see our Editorial Policies and the Editorial Policy Checklist.

Statistics

For all statistical analyses, confirm that the following items are present in the figure legend, table legend, main text, or Methods section.

n/a	Confirmed	
☐	☒	The exact sample size (*n*) for each experimental group/condition, given as a discrete number and unit of measurement
☐	☒	A statement on whether measurements were taken from distinct samples or whether the same sample was measured repeatedly
☐	☒	The statistical test(s) used AND whether they are one- or two-sided<br>*Only common tests should be described solely by name; describe more complex techniques in the Methods section.*
☒	☐	A description of all covariates tested
☐	☒	A description of any assumptions or corrections, such as tests of normality and adjustment for multiple comparisons
☐	☒	A full description of the statistical parameters including central tendency (e.g. means) or other basic estimates (e.g. regression coefficient) AND variation (e.g. standard deviation) or associated estimates of uncertainty (e.g. confidence intervals)
☐	☒	For null hypothesis testing, the test statistic (e.g. *F*, *t*, *r*) with confidence intervals, effect sizes, degrees of freedom and *P* value noted<br>*Give P values as exact values whenever suitable.*
☒	☐	For Bayesian analysis, information on the choice of priors and Markov chain Monte Carlo settings
☒	☐	For hierarchical and complex designs, identification of the appropriate level for tests and full reporting of outcomes
☒	☐	Estimates of effect sizes (e.g. Cohen's *d*, Pearson's *r*), indicating how they were calculated

Our web collection on statistics for biologists contains articles on many of the points above.

Software and code

Policy information about availability of computer code

Data collection	DP Controller (version 1.2.2.108, OLYMPUS); Zeiss LSM confocal 710; IncuCyte ZOOM; CFX Connect Real-Time System (BIO-RAD); Synergy 2 Multi-Mode Reader (BioTek); iBright CL1500 Imaging System (invitrogen by Thermo Fisher Scientific); Illumina NextSeq 550.
Data analysis	ImageJ (NIH); Prism (version 9.0, GraphPad); CFX Manager Software (BIO-RAD); Gen 5 software (BioTek); TopHat2 aligner; edgeR Bioconductor package; DAVID Bioinformatics Resources 6.8 (Laboratory of Human Retrovirology and Immunoinformatics); Western blot images were cropped by ACDSee12.0 software. Graphs for cell migration, colony formation, RT-qPCR, IHC and plots of KEGG pathway analysis were generated in Microsoft Excel software.

For manuscripts utilizing custom algorithms or software that are central to the research but not yet described in published literature, software must be made available to editors and reviewers. We strongly encourage code deposition in a community repository (e.g. GitHub). See the Nature Portfolio guidelines for submitting code & software for further information.

Data

Policy information about availability of data

All manuscripts must include a data availability statement. This statement should provide the following information, where applicable:

- Accession codes, unique identifiers, or web links for publicly available datasets
- A description of any restrictions on data availability
- For clinical datasets or third party data, please ensure that the statement adheres to our policy

All RNA-sequencing data generated have been deposited in the Gene Expression Omnibus under the accession code GSE193568. Source data related to all figures are included in this article. The other relevant data generated in this study are available from the corresponding author on reasonable request.

Field-specific reporting

Please select the one below that is the best fit for your research. If you are not sure, read the appropriate sections before making your selection.

☒ Life sciences ☐ Behavioural & social sciences ☐ Ecological, evolutionary & environmental sciences

For a reference copy of the document with all sections, see nature.com/documents/nr-reporting-summary-flat.pdf

Life sciences study design

All studies must disclose on these points even when the disclosure is negative.

Sample size	No sample-size calculations were performed. Sample size was determined according to previous experimental observations.
Data exclusions	No collected data were excluded.
Replication	All experiments were repeated with at least biological triplicates unless otherwise noted.
Randomization	Age- and littermate-matched mutant mice were randomly collected by genotype. Nude mice were randomized when they were used for tumor cell subcutaneous transplantation.
Blinding	Investigators were blinded to cell genotypes for RNA-sequencing performance and data analysis. For Western Blot and cell migration assay, investigators were blinded to cell genotypes for performance, imaging and analysis during biological replicate experiments. For mouse experiments, investigators were blinded to mouse genotypes for mouse phenotype observation, and blinded to tumor genotypes for tumor size monitoring.

Reporting for specific materials, systems and methods

We require information from authors about some types of materials, experimental systems and methods used in many studies. Here, indicate whether each material, system or method listed is relevant to your study. If you are not sure if a list item applies to your research, read the appropriate section before selecting a response.

Materials & experimental systems

n/a	Involved in the study	
☐	☒	Antibodies
☐	☒	Eukaryotic cell lines
☒	☐	Palaeontology and archaeology
☐	☒	Animals and other organisms
☒	☐	Human research participants
☒	☐	Clinical data
☒	☐	Dual use research of concern

Methods

n/a	Involved in the study	
☒	☐	ChIP-seq
☒	☐	Flow cytometry
☒	☐	MRI-based neuroimaging

Antibodies

Antibodies used	For Western blot:<br>FIP200 (D10D11) Rabbit mAb (Cell Signaling Technology, #12436); SQSTM1/p62 (D1Q5S) Cell Signaling Technology, #39749); Hamarin/TSC1 (D43E2) (Cell Signaling Technology, #6935); Phospho-ULK1 (Ser 757) (Cell Signaling Technology, #6888); ULK1 (Cell Signaling Technology, #8054); LC3B (1:2000, Cell Signaling Technology, #43566); Phospho-p70 S6 Kinase (Thr389) Antibody (Cell Signaling Technology, #9205); Atg5 (D5F5U) Rabbit mAb (Cell Signaling Technology, #12994); Atg7 (D12B11) Rabbit mAb (Cell Signaling Technology, #8558); Phospho-Stat3 (Tyr705) (D3A7) XP® Rabbit mAb (Cell Signaling Technology, #9145); Stat3 (124H6) Mouse mAb (Cell Signaling Technology, #9139); GAPDH (D16H11) XP® Rabbit mAb (Cell Signaling Technology, #5174), p70 S6 kinase a Antibody (C-18) (Santa Cruz Biotechnology, sc-230); Mouse Osteopontin/OPN Antibody (R&D Systems, MAB808); Monoclonal Anti-Vinculin (Millipore Sigma, V4505); Monoclonal Anti-β-Actin (Millipore Sigma, A5441); Anti-rabbit IgG, HRP-linked Antibody (Cell Signaling Technology, #7074); Anti-mouse IgG, HRP-linked Antibody (Cell Signaling Technology, #7076).<br><br>For immunohistochemistry:<br>CD31 (PECAM-1) (D8V9E) XP® Rabbit mAb (Cell Signaling Technology, #77699); Phospho-S6 Ribosomal Protein (Ser240/244) (D68F8) XP® Rabbit mAb (Cell Signaling Technology, #5364); Ki67 (Spring Bioscience M3060); Cleaved Caspase-3 (Asp175) (Cell Signaling Technology, #9661); Phospho-Stat3 (Tyr705) (D3A7) XP® Rabbit mAb (Cell Signaling Technology, #9145); Anti-Osteopontin (SPP1), mouse [LF-175] Antibody (Kerafast, ENH094-FP); Biotin-SP (long spacer) AffiniPure Goat Anti-Rabbit IgG (H+L) (Jakson ImmunoReearch Laboratories, Inc, 111-065-003). Alexa Fluor® 594 AffiniPure Donkey Anti-Rabbit IgG (H+L) (Jackson ImmunoResearch, Laboratories, Inc, #711-585-152) and Alexa Fluor® 488 AffiniPure Donkey Anti-Rat IgG (H+L)(Jackson ImmunoResearch Laboratories, Inc, #712-545-150)
Validation	Commercial per-validated antibodies were purchased from reputable sources (Cell Signaling Technology, Santa Cruz Biotechnology,

Validation	Millipore Sigma, R&D systems, Spring Bioscience, Kerafast and Jackson ImmunoResearch Laboratories), with validation data present on the manufacturer' websites as noted in the Methods section.

Eukaryotic cell lines

Policy information about cell lines

Cell line source(s)	HEK293T cell line was obtained from ATCC. 562 and 5864 cell lines were obtained from our lab.
Authentication	HEK293T cells were authenticated by short-tandem repeat profiling service. No further authentication has been performed since they arrived in our lab. We established 562 and 5864 cell lines by ourselves, they were not authenticated.
Mycoplasma contamination	All cell lines used in experiments were tested negative for mycoplasma contamination.
Commonly misidentified lines (See ICLAC register)	No commonly misidentified cell lines were used in this study.

Animals and other organisms

Policy information about studies involving animals; ARRIVE guidelines recommended for reporting animal research

Laboratory animals	Both male and female of Tsc1f/f;Scl-Cre, Tsc1f/f;Fip200f/f;Scl-Cre, Tsc1f/f;Fip200f/KI;Scl-Cre mice at 8-10 week-age were used with more than 98% C57BL/6 background. Female athymic nude mice at 6-8 week age were used in tumor cell transplantation.
Wild animals	No wild animals were used.
Field-collected samples	The study did not involve samples collected from the field.
Ethics oversight	Institutional Animal Care and Use Committee (IACUS) at University of Cincinnati

Note that full information on the approval of the study protocol must also be provided in the manuscript.
